# Incidental Findings with Genomic Testing: Implications for Genetic Counseling Practice

**DOI:** 10.1007/s40142-015-0075-9

**Published:** 2015-08-25

**Authors:** Myra I. Roche, Jonathan S. Berg

**Affiliations:** Department of Pediatrics and Genetics, School of Medicine, The University of North Carolina at Chapel Hill, 326A MacNider, Chapel Hill, NC 27599-7240 USA; Department of Genetics, The University of North Carolina at Chapel Hill, 120 Mason Farm Road, Chapel Hill, NC 27599-7264 USA

**Keywords:** Incidental findings, Secondary findings, Clinical sequencing, Genetic counseling, Medical actionability, Informed consent

## Abstract

This paper summarizes the current controversies surrounding the identification and disclosure of “incidental” or “secondary” findings from genomic sequencing and the implications for genetic counseling practice. The rapid expansion of clinical sequencing has influenced the ascertainment and return of incidental findings, while empiric data to inform best practices are still being generated. Using the North Carolina Clinical Genomic Evaluation by Next Generation Exome Sequencing (NCGENES) research project as an example, we discuss the implications of different models of consent and their impact on patient decisions.

## Introduction

The question of how to manage the broad range of genomic findings has emerged as one of the more contentious issues in the clinical application of genomic sequencing. In particular, there are concerns surrounding the inevitable generation of what could be considered “incidental findings,” frequently defined as a counterpoint to the primarily sought after diagnostic results and collectively described as the “incidentalome” [1••].

Historically used to classify research findings that arise during diagnostic testing [2], and routinely used in medical practice to describe additional findings unrelated to the indication for a particular evaluation, the colloquial meaning of “incidental” can imply something of lesser importance. This value judgment applies to some, but not all, incidental findings and alternative descriptors [3] have been suggested; each has its own promoters and detractors. Following its use by the Presidential Commission on Bioethical Issues, the term “secondary” is now preferred when such findings, unrelated to the diagnostic indication, are deliberately sought [4••].

Classifying secondary results in genetic testing is hardly new, and decisions to disclose their serendipitous discovery using genome-wide testing such as karyotype and microarray have been widely reported [5, 6]. The topic of secondary findings discovered via genomic sequencing has attracted responses from multiple disciplines including social scientists, clinicians, researchers, and bioethicists [7•, 8–12]. Paradoxically, while these variants are ubiquitous in the genome, their presence must be actively sought from among the vast number of other genomic variants in order to be identifiable and reportable.

In 2012, an American College of Medical Genetics and Genomic (ACMG) Working Group on Incidental Findings was assembled due to concerns of the ACMG that rapid expansion of clinical genome-scale sequencing could lead to heterogeneity in practices regarding incidental findings, and the perceived need to establish preliminary guidance for clinical laboratories. In 2013, the Working Group published the recommendation that laboratories should routinely analyze and report pathogenic variants from a specific set of genes when clinical diagnostic sequencing was ordered [13•]. They cautioned that patients should be forewarned that sequencing could reveal such findings but, once testing was ordered, the laboratory should analyze a small set of genes and report findings deemed to be “medically actionable” regardless of the proband’s phenotype or age.

Extrapolating on attempts to classify genetic tests by parameters such as clinical validity and clinical utility (http://www.cdc.gov/genomics/gtesting/ACCE/index.htm), the Working Group identified a list of 57 genes (later revised to 56) associated with conditions that were considered to reach a high bar of medical actionability. Allowing for differences in the population being sequenced and laboratories’ thresholds for asserting pathogenicity, the likelihood that a pathogenic variant will be found resulting in the disclosure of a medically actionable incidental finding has been estimated to be between 1 and 3 % in both research [14, 15•] and clinical studies [16]. These estimates have corresponded to predicted frequencies based on modeling [17]. It was anticipated that the minimal list would provide a focus for further discussions and would be modified in future renditions (Table 1).

**Table 1:** Conditions for which genes and variants are recommended for return of incidental findings in clinical sequencing as proposed by the ACMG

Hereditary breast and ovarian cancer
Li–Fraumeni syndrome
Peutz–Jeghers syndrome
Lynch syndrome
Familial adenomatous polyposis
*MYH*-associated polyposis
Von Hippel–Lindau syndrome
Multiple endocrine neoplasia type 1
Multiple endocrine neoplasia type 2
Familial medullary thyroid cancer
*PTEN* hamartoma tumor syndrome
Retinoblastoma
Hereditary paraganglioma–pheochromocytoma syndrome
Tuberous sclerosis complex
*WT1*-*related* Wilms tumor
Neurofibromatosis type 2
Ehlers–Danlos syndrome, vascular type
Marfan syndrome, Loeys–Dietz syndromes, and familial thoracic aortic aneurysms and dissections
Hypertrophic cardiomyopathy, dilated cardiomyopathy (including Fabry disease)
Catecholaminergic polymorphic ventricular tachycardia
Arrhythmogenic right-ventricular cardiomyopathy
Romano–Ward long QT syndrome types 1, 2, and 3, Brugada syndrome
Familial hypercholesterolemia
Malignant hyperthermia susceptibility

### Reaction to the ACMG Recommendations on Incidental Findings

The attempt to define the characteristics of conditions that would be sufficient to trigger professional obligations to identify and report secondary findings led to heated discussions of the ethical quandaries that ensue regarding their handling [18, 19]. The attempt to define categories of information within the vast scope of potential genomic findings has allowed genetic professionals to, more or less, coalesce around the general parameters and the idea of listing a minimal core group of genes, albeit without agreement on the particular genes on that list.

Many of the problematic aspects of the recommendations were acknowledged by the ACMG Working Group [13•] but sparked a number of critical commentaries nevertheless. Questions were raised as to whether or not the search for these variants was best viewed as being part of a professional’s obligations [20, 21] or if there were legal ramifications [22]. Other concerns included the potential extra work needed for variant interpretation and confirmation, and whether this effort would be compensated [23], the uncertain accuracy of genotypic predictions in populations in which familial segregation of the phenotype was absent [24], the technological gaps in sequence coverage [25], the potential harms of false positives to unwary patients and their relatives due to errors in the medical literature [26], and the apparent contradiction with the historical recommendations against testing asymptomatic children for adult-onset conditions [27•].

It also became apparent that the initial recommendations did not acknowledge the need for patients to have the opportunity to “opt out” of receiving such information, a position that is largely supported by genetics professionals [28]. A clarification of the 2013 recommendations published by the ACMG Board of Directors [29] addressed this concern by acknowledging the patient’s right “not to know” [30] and supported the ability of patients to opt out of categories of results.

### Provider Responsibilities, Informed Consent, and Patient Decisions

In addressing the difficult issues of how to account for individual contexts, the ACMG recommendations placed the responsibility as to how, when, and, if results should be communicated on a medical professional’s judgment [31]. A group of clinical laboratorians chose to emphasize the patient as the locus of decision making [32]; a stance echoed in research settings where participants are asked to pick and choose among the spectrum of potential genomic results. The choice and, therefore, the responsibility, to decide which results to learn and which results not to learn, are emblematic of a stronger focus on patient-centered decision making that is also affecting other areas of medical care. That being said, enabling a patient to make an informed decision remains the responsibility of the clinical provider, a role that has not changed with the expansion of testing from single gene tests to whole-genome sequencing.

If patients and participants are expected to routinely be asked to decide which secondary findings they want returned and which they do not, what preparation do they need to enable them to make informed decisions? A reflexive response to learn “everything” does not necessarily constitute an informed decision on the matter. The ability to opt out of learning secondary findings entirely is contingent on the recognition that the option exists and in the confidence that declining is a reasonable course of action. The decision to learn one potential finding but not another requires a broader understanding of the scope and magnitude of the universe of potential returnable results. More importantly, the level of understanding needed to decline to learn a potential result is even deeper [33•]. As the menu of gene variants that could potentially be returned grows, the depth of understanding needed for rational decision making also grows due to factors such as pleiotropy; complications often ignored and probably underestimated [34]. How coarsely or finely should the options be divided, and on which attributes can the spectrum of findings be reasonably categorized? How can a rational yet simple enough menu be devised, and communicated, to present understandable categories of clinically valid results, medically actionable or not? And, as variant classification evolves and new treatments become available, genes will inevitably shift from one category to another, further complicating future educational needs.

This patient-centric approach demands a robust informed consent process prior to clinical sequencing. It prompts questions of which information should be included and how it should be tailored to promote patient understanding. In the early days of patient sequencing (circa 2010), authors raised concern over the vast amounts of information they predicted would need to be communicated in order to obtain informed consent [35]. The time estimates to accomplish this supposedly Herculean task topped out at several hours [36], although these estimates were considered unrealistic [37] and, in practice, the approach has become more streamlined [38]. Indeed, in interviews with 29 genetic counselors and research coordinators who obtain consent, they reported spending an average of about 30 min by honing in on information relevant to the return of results [39].

Recommendations of elements to be included on a consent template have been made [40•, 41, 42] but consensus has not yet been achieved (Table 2). The need for a standard consent document template is highlighted by earlier studies of consent forms [43, 44]. To be fair, these forms had been created before enough experience had been gained to be able to achieve a consensus. Still, among sites engaged in translational clinical sequencing exploratory research, striking divergences and omissions were noted in the descriptions of potential diagnostic and secondary findings, the types of results to be returned or not, their inclusion in the medical record, and the role of participant preferences [45].

**Table 2:** Recommendations for informed consent for genomic sequencing (adapted from Ayuso, et al.; and the ACMG policy statement points to consider for informed consent for genome/exome sequencing)

Testing characteristics
Scope
Description of techniques
Results
Spectrum of returnable vs. non- returnable results
Likelihood of each
How and to whom results will be communicated
Risks, benefits, limitations, and testing alternatives
Special cautions about use in children
Management and choices to opt out of secondary findings
Voluntary participation
Confidentiality and privacy protections
Sample management
De-identification, sharing, and opt-out procedures
Possibility of re-contact with new information

The broad scope of genomic sequencing creates complex downstream implications depending on the types of results returned. Incomplete appreciation of these implications, and increasingly heightened scrutiny of these complexities, is a source of discrepancies between consent forms. Even standard items on consent forms, such as the expected risks and benefits, the protections for confidentiality, and the future use of the data, can have nuanced meanings. For example, although the voluntary withdrawal from research studies is an obligatory element of consent, it has been argued that this may be a disingenuous promise if results are placed into the medical record where they cannot be removed [46]. Despite the tendency to place different types of information into specific categories, segmentation of genetic information can also be problematic [47].

Which consent elements are considered essential may differ when applied to the potential for learning diagnostic, as compared to learning secondary, results. In particular, the assessment of the risks and benefits of learning diagnostic results by individuals searching for an explanation for their health condition could be expected to differ from the same person’s calculation when applied to learning a medically actionable, secondary finding. Alternatively, the decision to learn a medically actionable result may be seen as a form of empowerment by one whose medical condition is stable, as compared to another whose condition is progressive and degenerative; the latter may, instead, find the news distressing and overwhelming. Research participants and clinical patients can also have distinctly different expectations and thus reactions to information [48, 49]. Decisions about learning several kinds of secondary results that span a very broad spectrum of clinical utility could be expected to differ even more widely depending upon many contextual factors.

### Making Decisions About Secondary Findings is Complex: Do People Really Want Everything?

Given the complexity of potential secondary findings that could be identified through genome-scale sequencing, it was surprising to learn, as several studies concluded, that although genetic professionals found several extenuating factors that influenced their definition of a returnable result, non-professionals had apparently concluded that the obvious solution would be to just “ask for everything.”

Operating on the premise that to find out what people want is simply to ask them, early studies asked many different stakeholders; from clinical [50, 51] and research professionals [52], to those experienced with offering genetic testing [53], to IRB chairs, and to members of the public [54] and, in some cases, combinations of various stakeholders [55]. Since empirical data had yet to be collected about real decisions by people being asked to make them, this information gap was filled by a proliferation of studies that queried populations using hypothetical situations. Although severely limited in their widespread applicability, data from these surveys and focus groups raised conjectures about which kinds of attributes people were seizing on to make decisions. At the same time, beginning with the binning model developed by Berg et al. [7•, 56], clinical research groups expanded and experimented with different ways of categorizing secondary results including what would qualify as returnable and by what mechanisms they could or should be communicated [57•, 58, 59].

Genetic professionals have since come to a general consensus that a limited set of medically actionable results should routinely be returned, with the caveat that an individual’s “right not to know” be protected by the informed consent process [60•], perhaps through a formal opportunity to “opt out” of certain kinds of results. Disagreements between groups remained about how best to define and communicate the concept of “medical actionability” and how and if this category should be modified when minors are sequenced [61]. Recommendations about the complicating issues surrounding childhood testing have been made [62••] and qualifiers such as the age of onset of the condition and the child’s cognitive status are important [63]. It has been proposed that the identification and return of secondary findings, when identified in a child without a family history, are qualitatively different from the situation in which a child’s risk is already known by virtue of the presence of the condition in the family [19]. Christenhusz has advocated that disclosure of medically actionable variants be viewed as the default, allowing for some exceptions, such as the age and status of the patient [11]. Surveys of genetic professionals show increasingly more reservations about return of results when the characteristics of such findings veer further away from the highly penetrant, clearly pathogenic variants strongly associated with medically actionable conditions [28].

Data collected about these issues from parents, individuals with genetic disorders and lay people, on the other hand, tended to show more enthusiasm about the return of a broad spectrum of results [64]. Respondents discriminated between broad categories across the spectrum of conditions using characteristics such as the seriousness of the disorder, how likely it was to occur, the availability of effective treatments, and the age of onset.

Gathering data from eight focus groups that varied by age, gender, and professional status, Christenhusz found that, although the testing of minors was given special consideration, the mantle of parental responsibility was often the ultimate deciding factor [65••]. Even when they had second thoughts about learning information that could be ambiguous, fail to lead to any treatment, and be potentially harmful, most maintained that it was better to know than not to know. Respondents placed value on knowledge itself regardless of whether or not it led to action. They appeared to see only the forest of potential information and not the individual potentially risky trees and most welcomed “information” regardless of its accuracy, validity, or predicted potential for harm. Still, clinical actionability stood out as the benchmark against which all other characteristics were measured. Other scenarios were more contentious, such as evaluating children for adult-onset medically actionable conditions, identifying carrier status, and learning about a variety of other conditions that participants tended to view through a much wider lens of utility than that used by professionals.

This seemingly unanimous agreement that nearly all information was equally welcomed was congruent with reports from other studies that concluded that there was minimal harm, in some populations, in learning genetic information [66]. Taken together, these data might have meant that the difficult job of categorization would be far easier. Some remained uneasy, however, about the limited generalizability of responses from select populations [67, 68], and the inadequacy of psychological measures to detect subtler harms [69••]. There were also questions about how to qualify the degree of risk incurred in returning variants that did not meet a clinical actionability threshold and how to sort out which risks merited more caution than others. One concern that arises with regard to studies that report patient preferences to obtain even the “uncertain” genomic information is whether study participants truly understand the magnitude of uncertain findings that could be discovered. Furthermore, few studies have explored informational preferences related to the amount and quality of information—for example, when given a choice between receiving a handful of well-understood genomic findings or thousands of genomic findings with unknown clinical significance. An important challenge of eliciting patient preferences regarding whether to learn certain categories of information (and presumably not others) is to provide sufficient information to enable an informed choice. The process of setting patient preferences also requires balancing the efficiency of providing categorical choices versus the individualized customization of specific findings to be returned [70].

### Assessing Patient Preferences in Research and Clinical Practice

Studies of individuals experienced with sequencing in themselves or their children seemed to echo the prior reports of enthusiasm for genetic information. Reports from the ClinSeq project confirmed that most of the participant population looked to learn about all possible results [71]. This response might be expected, given the atypical characteristics of their select participants; a limitation the authors acknowledged. Parents in their study cited the obligation to learn everything possible about their children and, given their past experiences with their child’s rare and etiological mystifying condition, were confident they could withstand and incorporate any information regardless of its predictive ability [72]. Participants could distinguish how decisions might differ depending upon the category but many remained firm in their own desires to learn as much as possible.

More ambivalence was expressed during interviews conducted with sequenced patients with cancer and parents of children with undiagnosed conditions [73]. These participants expressed a wider variety of preferences but unanimously supported a central role for the patient in the decision-making process. Interviewees expected that additional findings would improve their lives by potentially explaining their diagnosis and they valued information as a way to prevent, or at least prepare, for the future. Even the potential to learn about untreatable conditions had the silver lining of being an opportunity to participate in research. Some participants reported that they would decline to learn information such as carrier status because acting on that information would be contrary to their religious beliefs. They also recognized that learning information is not always an unequivocally positive experience but can be burdensome and cause anxiety.

That so few respondents express anxiety specifically about the potential for genetic discrimination is not unusual; patients often fail to recognize it as a potential risk until after the person obtaining consent specifically raises it [74].

Even as evidence of patient ambivalence about learning secondary findings increased and the chorus advocating a slower pace grew louder [75, 76], the rate of clinical sequencing quickened. Data summarizing the sequencing experience of 200 patients who had been presented with an option of learning secondary findings were published [77•]. In this study, there appeared to be no ambivalence among those studied, as 93.5 % indicated that they desired secondary findings in any of four categories for which pathogenic variants were returned. Children, who made up 81 % of the population, were only eligible for results in the category of predisposition to early disease, while adults were eligible for three additional categories: carrier status, predisposition to later onset disease, and predisposition to cancer. Somewhat disturbingly, 15 % of those consenting for a child’s test requested results in categories for which they were not eligible, irrespective of the required pre-test genetic counseling and information in the consent form. Whether this result reflected a true desire for information on the part of parents, despite being counseled that it was not an option, or whether it indicated failure of clinicians and patients to understand the consent form, is not known. Among adults, 16 % declined at least one category, which was statistically different than the 4 % of declining parents/guardians. As with many candidates for sequencing, the children tested had limited life-spans with little expectation of being able to make autonomous decisions in the future. Such patients have been discussed as perhaps qualifying for a reasonable exception to the usual professional recommendations against testing for adult-onset conditions in children, but consensus has not been achieved on this point. Others have noted that when sequencing is done to explain a chronic health problem, individuals may not be ready or be able to think through the implications of learning secondary findings until after they learn their diagnostic results, even following a discussion about it [78].

## The NCGENES Experience with Secondary Findings

One barrier to describing the spectrum of results that could be learned from genomic sequencing is the sheer heterogeneity of potential information. Berg and colleagues developed a categorization scheme used in the North Carolina Clinical Genomic Evaluation by Next Generation Exome Sequencing (NCGENES) project [7•, 56, 58]. NCGENES is designed to investigate the performance of NGS technologies in the diagnosis of patients with suspected genetic disorders to determine their validity and best use in clinical care. The project, part of the NIH-funded Clinical Sequencing Exploratory Research (CSER) consortium, also seeks to evaluate the impact on participants of receiving diagnostic results, medically actionable secondary findings, and non-medically actionable secondary findings.

Defining the criteria by which conditions can be classified as medically actionable or, by contrast, non-medically actionable has been challenging [79] and discrepancies arise between what providers mean and what patients assume by this term. The term “medically actionable” focuses on actions that can be taken by a medical professional rather than the spectrum of actions that may be taken by patients regardless of their efficacy. The term “medically actionable” is narrowly defined in NCGENES as pathogenic or highly likely pathogenic variants that “confer a high likelihood of disease, for which knowledge of their presence allows medical interventions that can significantly reduce morbidity and mortality.”

Since most secondary findings have limited medical actionability, thereby leading to lack of consensus regarding their routine disclosure, the NCGENES project is specifically studying the potential benefits and harms of learning such information. Adult participants in NCGENES who are not cognitively impaired are randomized to either a group that learns diagnostic results and any medically actionable findings, or a group that is asked to decide, in addition, whether or not to learn any combination of six additional categories of non-medically actionable secondary findings. Both groups are followed to learn the impact of these results. Adults in the group randomized to make decisions about additional non-medically actionable findings are educated about the characteristics of each type including the implications of learning them and the eligibility of the results to be placed in the medical record (Table 3). Education occurs both by written information sent prior to their return of their diagnostic and medically actionable result visit and by an in-person discussion with a medical geneticist and genetic counselor at that visit. Importantly, this decision making occurs after the return of results visit, and participants are specifically asked not to make a decision at that time and are instead given the ability to initiate analysis at any subsequent time by contacting the study.

**Table 3:** Categories of secondary findings and return methods in the NCGENES project

Type of secondary finding		Returned by
A	GWAS risk SNPs	Telephone
B	Pharmacogenomics	Telephone
C	Carrier status	1 visit
D	APOE	1 visit
E	Mendelian disorders	1 visit
F	Severe neurodegenerative disorders	2 visits

In our preliminary experience with NCGENES participants who have been randomized to make a decision about non-medically actionable secondary findings, it appears that only a minority is requesting them. This result is in contrast to the expectation that most participants would request everything. It suggests that even when participants express an intention to learn secondary findings, these initial predictions may not reflect an unequivocal desire for them. It may be that, as in previous studies, participants are optimistic about the value of genetic information for future use (notwithstanding their limited utility at present), but if so, this optimism does not seem to translate into a desire to immediately learn the information. The endowment of information with an intrinsic power regardless of its expected utility may be more likely for people whose past searches for information to explain their condition have been unsuccessful. Another interpretation of these results is that if NCGENES participants have mixed feelings about learning additional findings, they can simply delay taking action rather than completely shutting the door on their options. This approach to requesting secondary findings is very different than the traditional informed consent model in which patients are required to make their decisions at the time of sequencing, as was the case in the results reported by Shahmirzadi and colleagues [77•].

A discrepancy between stated intentions and actual requests may be more likely to materialize when participants are not asked to decide at the time they consent for diagnostic sequencing. When given time to make their decision, participants have the chance to think through implications they may not have considered before, or to talk to family members or others who were not available at the time of consent. When given space to make their decision, removed from the sphere of influence of a health care professional, participants may feel more freedom to, at the very least, delay the decision if they have doubts. And when empowered to take the first step to initiate the analysis, participants may be convinced that declining is a reasonable option. It may also be expected that these decisions assume lesser importance as time goes by and regular life resumes.

In contrast, when the decision to opt in or out of learning secondary findings is made at the time of consent, participants’ assumptions about the process may lead them to opt in, just in case. In research studies, participants may think their results have already been generated and are known to the research team, or they may assume that by declining information they will, in some way, hinder the research. In a clinical setting, patients may view opting out as potentially jeopardizing their ability to learn information that one day may be important. In either case, most individuals have limited experience in being invited to decline a medical test or to assess its risks, and there may be social stigma attached to declining information, regardless of its value. The opportunity for cost-free testing can also be a powerful incentive.

### Conclusions and Future Goals

Much of the current controversy over the management of secondary findings in genome-scale sequencing, whether in a research or clinical context, revolves around the perceived differences in the roles and responsibilities of professionals and the rights and preferences of participants. Finding the balance between the appropriate degree of professional guidance and individual choice will require more than vigorous commentary and the reporting of subjective data on hypothetical preferences, but will require empiric data on actual decisions and their outcomes. Traditional modes of informed consent and genetic testing will need to evolve in order to accommodate the increasing complexity of genome-scale sequencing. If, as some anticipate, the role of genomic sequencing in clinical care expands, to become an integral part of medical care, then the roles and responsibilities of clinicians and the rights and preferences of patients may assume a longitudinal nature in which decisions to query information will be made over the course of an individual’s life and not necessarily at the moment of consent for sequencing.

Several important tasks remain. Attributes that are central to patients’ decisions to learn secondary findings need to be identified. For example, Reiger et al. have conducted a discrete choice experiment to quantify participant preferences by asking them to make trade-offs to rank the relative importance of attributes such as lifetime risk, treatability, seriousness, and cost [80•, 81]. Alternative models of consent and disclosure are being piloted, and staged versions of both may help scale up the genetic counseling process [40•, 57•, 82]. The development of more sensitive tools to identify and track long-term effects of learning genetic information could help define subtler effects associated with better or worse long-term adjustment [69••, 83]. Several groups, such as those in the CSER Consortium and the Electronic Medical Records and Genomic (eMERGE) Network, are collecting data to help inform these tasks.

Finally, there is an urgent need to develop educational strategies to enhance the way people make informed decisions that streamline, yet complement, the genetic counseling process [67]. Electronic decision aids [84, 85] and other tools, both electronic and not, can lay a foundation of knowledge, but the importance of interpersonal dialog to help people reach complex decisions that are right for them should not be underestimated nor discarded. The discrepancies between consent form content and patient comprehension illustrate its importance in promoting understanding, patient autonomy, and shared decision making [86].

As clinical sequencing segues into other populations, such as newborn screening, [87, 88] our definitions and understandings of the risks and benefits of learning genomic findings will evolve, forcing the development of new models of education and counseling. In the era of personalized genomic medicine, genetic counseling has the opportunity to become even more effective and valuable if it can adapt without losing the personalized essence of what it can accomplish.

If it is true that “stories trump numbers and relationships trump stories” [89], educational strategies that touch both the cognitive and the emotional chords in the decision-making process by helping patients forecast their short- and long-term emotional responses to their decisions will help keep genetic counseling relevant regardless of what genomic testing looks like in the future.
